# Over expression of the selectable marker blasticidin S deaminase gene is toxic to human keratinocytes and murine BALB/MK cells

**DOI:** 10.1186/1472-6750-4-29

**Published:** 2004-12-02

**Authors:** Fernanda Mara Bento, Daniela Takeshita, Chester Bittencourt Sacramento, Tamara Rocha Machado, Monica Beatriz Mathor, Adriana Karaoglanovic Carmona, Sang Won Han

**Affiliations:** 1 Department of Biophysics & Center for Gene Therapy, UNIFESP – EPM, São Paulo, SP, Brazil; 2 Instituto de Pesquisas Energéticas e Nucleares – CNEN, São Paulo, SP, Brazil

## Abstract

**Background:**

The blasticidin S resistance gene (*bsr*) is a selectable marker used for gene transfer experiments. The *bsr *gene encodes for blasticidin S (BS) deaminase, which has a specific activity upon BS. Therefore, its expression is supposed to be harmless in cells. The work reported on herein consisted of experiments to verify a possible toxicity of *bsr *on mammalian cells, which include several cell lines and primary cultures.

**Results:**

Murine keratinocyte BALB/MK and human primary keratinocyte cells transduced with the retroviral vector LBmSN, which has an improved expression system of *bsr*, namely *bsrm*, died in five days after the transduction. Meanwhile the control vector LBSN, which expresses *bsr*, did not provoke cell death. The lethal activity of *bsrm *was observed only in human keratinocytes and BALB/MK cells among the cell types tested here. Death appears to be mediated by a factor, which is secreted by the BALB/MK transduced cells.

**Conclusion:**

By our study we demonstrated that the expression of *bsrm *gene is toxic to human keratinocytes and BALB/MK cells. It is likely over expression of BS deaminase gene is responsible for the death.

## Background

Blasticidin S (BS) is a nucleoside antibiotic isolated from *Streptomyces griseochromogenes*, and has been used as a fungicide against rice blast disease [1]. BS inhibits protein synthesis in both prokaryotes and eukaryotes [1]. Later, a gene that provides resistance against BS (*bsr*) was isolated from *Bacillus cereus *K55-S1 strain [2,3]. The *bsr *gene possesses only 420 bp [4] and codes for an enzyme of 15 kDa, which is usually present in its dimer form [5]. The enzyme acts upon BS converting it into a deaminohydroxy derivative [5,6], thus it is named BS deaminase.

BS antibiotic is highly toxic to mammalian cells; even 2 μg/ml is enough to kill HeLa cells in a few days. However, the cells transfected with *bsr *could resist against BS at several fold higher concentrations [7]. Hence, the BS/*bsr *pair has been used as an efficient selection system for gene transfer experiments in many different cell types.

Recently, we reported that some modifications introduced into the non-coding regions of the *bsr *gene (*bsrm*) resulted in an increase (several fold) of *bsr *gene expression, and consequently, NIH3T3 cells transduced by retroviral vectors could be selected with higher concentrations of BS in just a few days [8]. Even with such extensive use of the *bsr*/BS selection system, no side effects in response to *bsr *gene expression have been observed.

Using the murine keratinocyte cell line BALB/MK [9] and human primary keratinocytes, we report here a surprising death of the keratinocytes provoked by the expression of *bsrm*. A detailed investigation about the death of keratinocytes, which was mediated by an unknown molecule and secreted by the BALB/MK cells transduced with LBmSN, is discussed below.

## Results

### Sensitivity of BALB/MK cells to BS

To determine the range of BS concentrations and the time required for BALB/MK cell death to occur, 1 × 10^4 ^cells/well were incubated in a 24 well plate and 2 days later the media were replaced with a fresh one containing various concentrations of BS for 9 days. A BS concentration of at least 2 μg/ml was necessary to kill the cells in 5 days. At concentrations of BS greater than 8 μg/ml the majority of cells died within 3 days.

### Effect of bsrm gene and BS on BALB/MK cells

**Figure 1:**
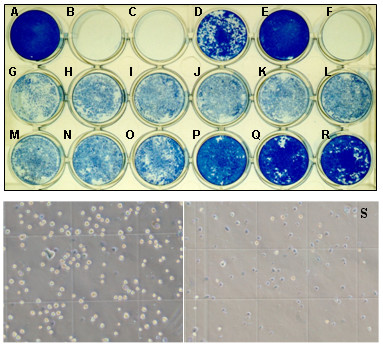
**Effect of BS upon the BALB/MK cells transduced with LBmSN **On day 1, BALB/MK cells were seeded on a 24-well plate at 1 × 10^4 ^cells/well, and 1 × 10^6 ^cells of the virus producing cell lines PA317/LBmSN (2 × 10^5 ^cfu/ml) and PA317/LXSN (5 × 10^6 ^cfu/ml) were seeded in 25 cm^2 ^flasks. On day 2, the media of PA317 cell clones were replaced with EMEM without EGF. On day 3, the media of PA317 cells containing virus were harvested, centrifuged at 10000 rpm per 1 min in Eppendorf centrifuge. The media of BALB/MK cells were replaced with 0.5 ml of virus solution and supplemented with 5 ng/ml EGF and 8 μg/ml Polybrene. On day 4, the media were replaced with fresh EMEM in the following conditions. After 12 days, the cells were stained with Coomassie Blue and photographed. The results are representative of at least three experiments, which gave essentially the same results. (A) BALB/MK; (B) BALB/MK + 500 μg/ml G418; (C) BALB/MK +  8 μg/ml BS; (D) BALB/MK/LXSN + 500 μg/ml G418; (E) BALB/MK/LXSN; (F) BALB/MK/LXSN + 8 μg/ml BS; (G) BALB/MK/LBmSN; (H) BALB/MK/LBmSN + 500 μg/ml G418; (I) BALB/MK/LBmSN + 0.05 μg/ml BS; (J) BALB/MK/LBmSN + 0.1 μg/ml BS; (K) BALB/MK/LBmSN + 0.2 μg/ml BS; (L) BALB/MK/LBmSN + 0.5 μg/ml BS; (M) BALB/MK/LBmSN + 1 μg/ml BS; (N) BALB/MK/LBmSN + 2 μg/ml BS; (O) BALB/MK/LBmSN + 4 μg/ml BS; (P) BALB/MK/LBmSN + 8 μg/ml BS; (Q) BALB/MK/LBmSN + 16 μg/ml BS; (R) BALB/MK/LBmSN + 32 μg/ml BS(S) Five days after the LBmSN transduction, the cells were detached with trypsin and stained with Trypan blue. Left and right sides represent BALB/MK (control) and BALB/MK/LBmSN cells respectively.

To verify the effect of *bsrm *gene in BALB/MK cells, the cells were transduced with the LBmSN retroviral vector, which expresses the *bsrm *and *neo*^*R *^genes, and then incubated with BS. The *bsrm *gene was obtained by modifying the *bsr *gene at a non-coding region and, consequently, there was no alteration of the amino acid sequence [8]. The addition of BS at concentrations of ≤ 4 μg/ml or 500 μg/ml of G418 to cell media resulted in the death of all transduced BALB/MK (Figure 1G,1H,1I,1J,1K,1L,1M,1N,1O). In contrast, the transduced cells incubated with BS at concentrations of ≥ 8 μg/ml reached confluence within 12 days of culture (Figs. 1P,1Q,1R). As an experimental control, BALB/MK cells transduced with the LXSN vector (which expresses only the neo^R ^gene) [10] were incubated in the presence or absence of 500 μg/ml of G418. As expected, the cells reached partial or complete confluence within 12 days (Figs. 1D and 1E), as also observed with the non-transduced BALB/MK cells (Figure 1A).

**Figure 2:**
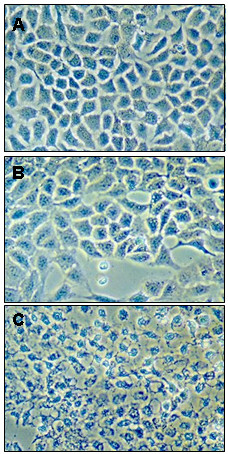
**Phase contrast microscopic appearance of the BALB/MK cells transduced with LBmSN **The cells were cultured and transduced as described above. After 8 days of the transduction, the cells were photographed (X 200). The results are representative of at least five experiments, which gave essentially the same results. (A) BALB/MK; (B) BALB/MK/LBmSN + 8 μg/ml BS; (C) BALB/MK/LBmSN

BALB/MK cells not expressing the *bsrm *gene died in the presence of 8 μg/ml of BS (Figs. 1C and 1F) as expected. However, the aspect of the BALB/MK cells which died after the expression of *bsrm *(Figs. 1G,1H,1I,1J,1K,1L,1M,1N,1O) was different from the aspect of the non-transduced cells (Figs. 1B and 1C) or cells transduced with LXSN (Figure 1F) which were killed by antibiotics alone. BALB/MK cells which died after expressing the *bsrm *gene had a reduced cell volume when compared with normal BALB/MK or transduced BALB/MK incubated with 8 μg/ml BS (Figure 2). Additionally, the cells, which died after *bsrm *expression, remained on the plate after washing with PBS, whilst the cells killed by the antibiotics, BS or G418, were easily removed after washing with PBS (Figure 1). The death of BALB/MK cells was confirmed by staining with Trypane blue (Figure 1S).

Removal of BS from the medium of the cells selected with 8 μg/ml BS resulted in cell death within a week (not shown). Thus, the BS antibiotic counteracted the death effect of *bsrm *and therefore has a vital role to BALB/MK cells transduced with LBmSN.

### Effects of cell density and virus concentration upon induction of BALB/MK cell death

Based on the above observations two variables were analyzed to assess their influences on the cell death: virus concentration and cell density. The total BALB/MK cells, transduced and non-transduced ones, were seeded at 1 × 10^3 ^to 4 × 10^4 ^cells in 25 cm^2 ^flasks and the cell death was monitored by optical microscope. The flasks containing a higher number of cells had faster cell death, even if the ratio of virus per cell was maintained constant in all flasks (Table 1). However, the absolute number of *bsrm*-transduced BALB/MK cells was higher in the flasks with higher number of seeded cells; consequently cell death was directly related to the presence of the number of *bsrm*-transduced cells.

**Figure 3:**
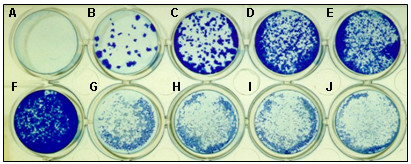
**The effect of the concentration of the virus LBmSN upon BALB/MK cells **The cells were cultured and transduced as described in Figure 1, however the virus concentration in each plate varied as follows. After 10 days of the transduction, the cells were stained with Coomassie Blue and photographed. The results are representative of at least three experiments, which gave essentially the same results. (A) BALB/MK + 8 μg/ml BS; (B) BALB/MK + 10^2 ^cfu of LBmSN + 8 μg/ml BS; (C) BALB/MK + 10^3 ^cfu of LBmSN + 8 μg/ml BS; (D) BALB/MK + 10^4 ^cfu of LBmSN + 8 μg/ml BS; (E) BALB/MK + 10^5 ^cfu of LBmSN + 8 μg/ml BS; (F) BALB/MK; (G) BALB/MK + 10^2 ^cfu of LBmSN; (H) BALB/MK + 10^3 ^cfu of LBmSN; (I) BALB/MK + 10^4 ^cfu of LBmSN; (J) BALB/MK + 10^5 ^cfu of LBmSN

The virus concentration used to transduce BALB/MK cells was evaluated by infecting the cells with 1 × 10^2 ^to 1 × 10^5 ^cfu (colony forming units) of LBmSN vector. The transduced cells incubated with 8 μg/ml of BS produced resistant colonies proportionally to the used virus concentration (Figure 3). LBmSN-transduced BALB/MK cells that did not undergo selection died at all virus concentrations (Figs. 3G,3H,3I,3J), although the cells transduced with 1 × 10^5 ^cfu died 2 days earlier than the cells transduced with 1 × 10^2 ^to 1 × 10^3 ^cfu. This is an extremely important observation since even in those wells containing less virus than cells (Figs. 3G and 3H) cell death occurred simultaneously in each cell. This result suggests the existence of intercellular signaling of death. To confirm this hypothesis, we seeded the LBmSN transduced and non-transduced BALB/MK cells together with or without BS (Table 2). A clear induction of death in BALB/MK cells by the BALB/MK cells transduced with LBmSN was observed. Changes of cell morphology in each colony occurred within 5 days, as was seen in all experiments.

**Figure 4:**
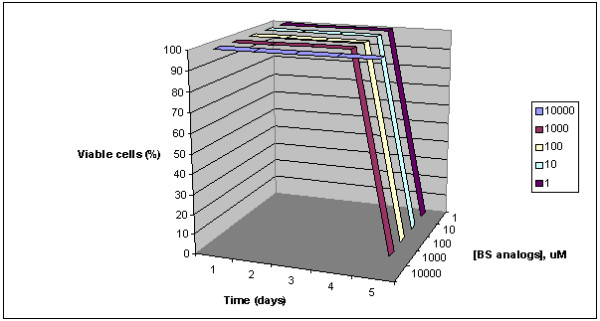
**Effect of analogs of BS upon BALB/MK cells transduced with LBmSN **The cells were cultured and transduced with LBmSN as described in Figure 1. On day 4 the media were replaced with a fresh one and BS-analogs were added at the indicated concentrations. This time is indicated as day zero in the figure.

To investigate whether the death signaling is mediated by a secreted factor, we tested the supernatant of the BALB/MK cells transduced with LBmSN on BALB/MK cells (Figure 4). A just two-day old medium was sufficient to induce death of normal BALB/MK cells. This result indicates that cell death was mediated by a soluble factor, secreted by the LBmSN-transduced cells, acting on both transduced and non-transduced BALB/MK cells. This factor we denominated DOKEB (Death factor Obtained from Keratinocytes Expressing Bsrm) to ease our discussion. DOKEB appears to be secreted only by LBmSN-transduced BALB/MK cells, because the 5 day old-medium from the LBmSN-transduced NIH3T3 cells had no death activity upon BALB/MK or NIH3T3 cells (data not shown).

### Effect of bsrm on mammalian cell lines

The lethal effect provoked by *bsrm *was firstly observed in the murine keratinocyte cell line BALB/MK as described above. To verify this lethal effect in other cell types, we chose the cells originated from the skin or epithelium (NIH3T3, HeLa, LISP-A10, LISP-E11, HCT-8 and B16F10), because of the origin of the BALB/MK cells [11]. In addition, the rat vascular smooth muscle cells, which are useful for gene therapy experiments [12], were also tested.

Until 7 days post-infection none of the above cells, which were transduced with LBmSN retroviral vector, did not die, whereas the control cell line BALB/MK died 5 days after the transduction (not shown). As the viral transduction rate is essential to analyze the possible death effect by the expression of *bsrm*, the cells were transduced with a ten-fold higher number of viruses than cells. Even in such conditions no cell types suffered with the expression of *bsrm*.

To ensure the transduction and expression of *bsrm *in the cells, those transduced cells were selected with 8 μg/ml of BS from the non-transduced ones that die in 4 days. The selected cells were distributed to two plates, and in one plate the initial concentration of BS was maintained and in from the other plate the BS was removed. During the 7 days of observation no death was observed in both plates (not shown), which confirm the previous result that the *bsrm *gene is not lethal to those cell types.

We also compared death activity of LBmSN and LBSN, which express *bsrm *and *bsr *respectively, on BALB/MK and NIH3T3 cells. Transduction of LBSN vector on BALB/MK or NIH3T3 cells did not cause cell death; meanwhile LBmSN caused cell death as expected (Table 3). In the presence of BS both cell lines transduced with LBmSN or LBSN did not die, which is a demonstration of BS deaminase gene expression, and also protection of the LBmSN transduced BLAB/MK cells against death as seen before. These results infer that over expression of BS deaminase gene could be responsible for the death of BALB/MK cells expressing *bsrm*.

### Effect of the BS analogs on BALB/MK cells

The analogs of BS, cytidine, 5'-deoxycytidine, uridine and 5'-deoxyuridine, were tested in the culture of the BALB/MK cells transduced with LBmSN at 1 μM to 10 mM concentrations (Figure 4). Interestingly all analogs with 10 mM protected the transduced cells during 5 days of observation. Changing the medium with a fresh one containing 10 mM of each analog at every five days, the cells could be maintained alive for several passages (not shown).

### Effect of the bsrm gene and BS upon human keratinocytes

**Figure 6:**
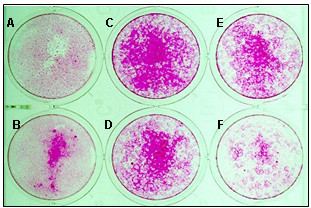
**Effect of BS upon the human keratinocytes transduced with LBmSN vector **On day 1, the irradiated PA317/LBmSN cells (30 Gy) and the human keratinocytes were seeded together at 3.3 × 10^5 ^and 5 × 10^4 ^cells per well respectively in a 12 well plate. Two days later, the medium was replaced with a fresh one containing the indicated concentrations of antibiotics. The cells were stained with Rhodamine B after 8 days. (A) no antibiotic; (B) 800 μg/ml G418; (C) 8 μg/ml BS; (D) 12 μg/ml BS; (E) 16 μg/ml BS; (F) 32 μg/ml BS

The transduced human keratinocytes, which were modified with the virus producing cell clone PA317/LBmSN as a feeder-layer, did not grow during 8 days of culture in the absence of BS (Figure 6). Incubating the transduced keratinocytes with BS at 0.05 to 2 μg/ml, which are tolerant concentrations by the cells, also resulted in the absence of cell growth (not shown). However, the presence of 8 μg/ml of antibiotic, in the medium, which is a lethal concentration for the cells, resulted in the formation of many keratinocyte colonies. The number of these BS resistant colonies decreased as the BS concentration increased (Figure 6).

**Figure 7:**
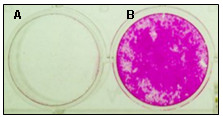
**Effect of BS upon the keratinocytes transduced with LBmSN and selected with BS **The keratinocytes were cultured and selected with 8 μg/ml BS as described in Figure 5. When the keratinocytes reached 70 % confluence, the cells were divided into two parts and transferred to 2 wells of a 12-well plate that contained the irradiated NIH3T3 cells. In one well the cells were maintained without BS (A) and in another with 8 μg/ml BS (B). The cells were stained with Rhodamine B after 10 days.

The BS selected cells could be maintained alive even with the expression of *bsrm *if the medium is replaced every two days with a fresh one containing the initial concentration of BS. Nevertheless, if those cells were seeded on a new plate without BS they died in few days (Figure 7). This result confirms the vital role of BS in *bsrm *expressing keratinocytes, as was observed with murine keratinocyte BALB/MK cells.

The vector LBmSN also expresses *neo*^*R*^, which provides resistance against geneticin. However, the transduced keratinocytes died after the incubation with 500 μg/ml of geneticin (Figure 6) even if the antibiotic was neutralized by aminoglycoside phosphotransferase. This result corroborates previous data that in keratinocytes the expression of *bsrm *gene leads to cell death. The keratinocytes transduced with the LXSN vector [10], which was used to construct the LBmSN vector, presented normal growth reaching complete confluence within 8 days (not shown).

## Discussion

Here we report a surprising death caused by the transduction of the murine keratinocyte cell line BALB/MK cells with the retroviral vector LBmSN. This vector expresses *bsrm *which is a modified form of *bsr *only in the non-coding region; consequently both genes express the same BS deaminase. As the vector LBSN, which expresses bsr much lower than LBmSN [8], did not kill the cells an over expression of bsr could be responsible for the death. However, we can not discard other possibilities caused by interactions of the bsrm gene product (mRNA or protein) with intracellular molecules.

In some cases the Moloney murine retrovirus can cause cell death [13]. However in our case the control retroviral vector LXSN, which was used to construct LBmSN and LBSN does not carry any viral genes [8], did not induce cell death in the same culture conditions (Figure 1). Additionally the wide range of viral concentrations and cell densities tested here (Table 1 and Figure 3) did not affect cell death. These last results corroborate the above conclusion that the death of BALB/MK cells was caused by the expression of *bsrm*.

An interesting phenomenon of the death of BALB/MK cells expressing *bsrm *was that those cells can be rescued if a lethal concentration of BS (8 μg/ml to 32 μg/ml of BS were tested) is added in the medium before three days after transduction. It was a paradox, since the toxic antibiotic, BS, was able to rescue the murine keratinocyte BALB/MK induced to die by the apparently inoffensive bacterial *bsrm *gene.

BS-rescuing process of the *bsrm*-transduced keratinocytes could be understood as a consequence of inhibition of the death factor (DOKEB) production by BS. Because those transduced keratinocytes could be maintained for long period (at least two months) simply by changing the medium every two days for a fresh one containing 8 μg/ml of BS (not shown). However, we do not know if the inhibition of DOKEB production is caused by the inhibition of protein synthesis by BS or just occupation of the active site of the BS deaminase by BS, and consequently inhibiting the binding of the first target molecule which is a responsible for the induction of the death process. We believe more in the last explanation, because if the analogs of BS were added to the medium at concentrations higher than 1 mM, cell death can be avoided (Figure 4). The requirement of higher concentration of the analogs of BS than BS, which requires only 19 μM to protect the *bsrm *expressing BALB/MK cells, is likely due to the low affinity of analogs for BS deaminase as expected [5].

Even though we have demonstrated that the expression of the *bsrm *gene in BALB/MK is lethal, the bsrm/BS selection system can still be used in keratinocytes. During the selection of LBmSN-transduced keratinocytes, the initial concentration of BS in the medium should determine a strict range of LBmSN transduced keratinocytes, which express not more and not less than certain levels of *bsrm *gene (Figure 6). Thus, for survival of those transduced cells AND selected with 8 μg/ml BS, the medium should be changed every 2 days to maintain active BS concentration in the medium; otherwise, the low BS concentration will allow the synthesis of DOKEB and in turn trigger the death mechanism. The BALB/MK cells transduced with LBmSN and selected with 8 μg/ml of BS should not be challenged with concentrations much higher than those used, since the cells expressing higher levels of *bsrm *gene should have died during the previous selection. Thus, the selected BALB/MK cells exist in a precarious situation where either apoptosis or necrosis can be easily activated at any unfavorable moment.

We evaluated if the lethal effect provoked by *bsrm *occurs exclusively in BALB/MK cells or if this phenomenon is general for all cell types. In this study we included normal human primary keratinocytes and several cell lines. The human keratinocytes are resistant to BS at concentrations lower than 2 μg/ml and at concentrations higher than 8 μg/ml the cells die within 5 days (not shown). However, the keratinocytes transduced with LBmSN behaved in an opposite way. In the absence or presence of BS at low concentrations (lower than 2 μg/ml) the transduced cells died, as it was observed with the BALB/MK cells transduced with LBmSN. Additionally, the protection against the death of the BALB/MK cells expressing *bsrm *by the addition of a lethal concentration of BS was also observed with the human keratinocytes expressing *bsrm *(Figs. 6, 7). These results indicate that the death process triggered by the expression of *bsrm *in keratinocytes should follow the same way.

Interestingly the lethal effect provoked by *bsrm *appears to be specific to keratinocytes, because none of the cell types tested here died in our experimental conditions, except for the human and murine keratinocytes. As the gene transfer and expression of *bsrm *are an essential step to access the lethal effect of *bsrm*, an alternative strategy used to verify the gene expression and its effect was selecting the transduced cells with 8 μg/ml BS and exposing the cells to a fresh medium without BS. Even in such conditions the *bsrm *gene did not cause any morphological alterations to those cell types, whereas the BALB/MK cells and the human primary keratinocytes transduced with LBmSN and selected with BS died in a few days after removal of the antibiotic (Figure 7). Therefore, we conclude that the lethal activity of *bsrm *is specific to those keratinocytes.

The analysis of DOKEB through exclusion molecular chromatography showed that the factor has a molecular weight equivalent of two amino acids (not shown). Therefore, DOKEB should not be BS deaminase, or even any protein. Further purification and molecular analysis are in progress.

In this study we demonstrated only in *vitro *that the expression of the reporter gene *bsrm *has a lethal effect on keratinocytes. However as most of the gene therapy experiments using keratinocytes are carried out *ex vivo *with retroviral vectors, our finding has a very important meaning. Because the cells transduced with retroviral vector carrying *bsrm *and selected with BS can survive until the antibiotic is maintained in the medium, but when those cells are returned to the own organism, which has no BS in it, DOKEB will be produced and can provoke serious lesion in the body.

By this study we also point out the danger of using heterologous genes, in particular those isolated from the microorganisms, in gene transfer and gene therapy experiments without proper controls.

## Conclusions

We demonstrated in this study that the expression of the reporter gene *bsrm *has a lethal effect on the murine BALB/MK cell line and human primary keratinocytes. It is likely over expression of the BS deaminase gene is responsible for the death. The death appears to be mediated by a factor, which is secreted by the BALB/MK transduced cells. By this study we point out the danger of using heterologous genes, in particular those isolated from the microorganisms, in gene transfer experiments without proper controls.

## Methods

### Retroviral vectors

The retroviral vectors used in the present study are based on the Moloney murine leukemia virus: LXSN [10], LBSN [8] and LBmSN [8]. The letters L, X, S, N, B, Bm of those vectors represent retroviral LTR promoter, cloning site, promoter of simian virus SV40, neomycine resistance gene (neo^R^), bsr and bsrm, respectively. The LBSN and LBmSN vectors were constructed inserting the *bsr *and *bsrm *genes into the Hpa I site of LXSN, which is located in the cloning site [8].

### Cell line culture

The amphotropic retrovirus producing cell clones PA317/LBmSN, PA317/LBSN and PA317/LXSN [8], the murine fibroblast NIH3T3, HeLa, the human colorectal carcinoma cell lines LISP-A10 and LISP-E11 [14] were cultured in Dulbecco's modified Eagle medium (DMEM) with high glucose (4.5 g/ml), supplemented with 2 mM glutamine, 200 U/ml penicillin, 200 μg/ml streptomycin and 10 % fetal bovine serum (FBS) (InVitrogen, São Paulo, Brazil) at 37°C in a humidified atmosphere with 5 % CO_2_. The mouse fibroblast CCL-92 (ATCC) was cultured as above, except that the FBS was replaced with the bovine calf serum (InVitrogen, São Paulo, Brazil). For the culture of the murine keratinocyte BALB/MK cells (kindly provided by Dr Stuart A. Aaronson, The Derald H. Ruttenberg Cancer Center, New York, NY), EMEM (Biofluids, Rockville, MD) containing 0.05 mM CaCl_2_, 10 ng/ml of EGF and 10 % FBS was used.

The human colorectal carcinoma cell line HCT-8 and the murine melanoma cell line B16F10 [15] were cultured in RPMI 1640 (InVitrogen, São Paulo, Brazil) supplemented with 0.2 % NaHCO_3_, HEPES 10 mM, pH 7.3, 40 μg/ml garamicine and 10 % FBS at 37°C in a humidified atmosphere with 5 % CO_2_.

### Rat primary smooth muscle cell culture and viral transduction

A primary culture of rat smooth muscle cells was prepared as previously described [12], digesting the Wistar isogenic rat aortas enzymatically. These cells were characterized immunocytochemically using antibodies against α-actin (Boehringer Mannheim, São Paulo, Brazil) for SMC positive staining and von Willebrand factor (Boehringer Mannheim, São Paulo, Brazil) for SMC negative and endothelial cell positive staining [12]. Only early-passage smooth muscle cells were exposed for 24 h to virus harvested from PA317/LBmSN cells for a period of 24 h in the presence of Polybrene (8 μg/ml, Sigma)

### Transduction of mammalian cell lines with retroviral vectors

The target cells were seeded on a 24 well plate at 1 × 10^4 ^cells per well with an appropriate medium as mentioned above. In parallel, 1 × 10^6 ^of virus producing cells (PA317/LBmSN, PA317/LBSN or PA317/LXSN) were seeded in a 25 cm^2 ^flask. After 24 h, the media from the target cells and the virus producing cells were replaced with a fresh one used for target cells. On the next day, the media of the target cells were replaced with 500 μl of the virus solution collected from the supernatant of the PA317/LBmSN cell culture and filtered in 0.45 μm syringe filter. Polybrene was added to the virus solution at the final concentration of 8 μg/ml. One day after the infection, the media were replaced with a fresh one, maintained in the CO_2 _incubator and the cells were observed using a microscope everyday.

In parallel, after two days of the infection, a new set of the transduced cells was split and only 1/10 part of the cells was maintained in the same well. The BS antibiotic was added to the wells at concentrations between 0 to 32 μg/ml. When the cells reached confluence they were split and transferred to two wells of a 12-well plate. To one well, BS was added at the concentration used for selection, and another well was maintained without BS. The cells were observed under the microscope everyday.

### Human primary keratinocytes culture and viral transduction

Normal human keratinocytes from healthy adult volunteers were obtained by biopsy, cut in small pieces and incubated in a trypsin solution (0.05 %) containing 0.01 % EDTA at 37°C for 3 h under constant agitation. Every 30 min the detached cells were transferred to a new 75 cm^2 ^flask containing 2 × 10^6 ^cells of the irradiated CCL-92 cells (60 Gy) as a feeder-layer. The medium used for the culture of the keratinocytes was composed of DMEM and Ham's F12 (2:1) containing 10 % FBS, 4 mM glutamine, 50 IU/ml streptomycin- penicillin, 0.18 mM adenine, 5 μg/ml insulin, 5 μg/ml transferrin, 0.4 μg/ml hydrocortisone, 0.1 nM choleric toxin and 20 pM triiodotyronin [11]. The medium was replaced every 2 to 3 days with a fresh one containing 10 ng/ml EGF. The cells were maintained in a humidified atmosphere with 5 % CO_2_.

For retroviral transduction, the packaging clones PA317/LBmSN and PA317/LXSN were irradiated with 30 Gy and used as a feeder-layer for the prepared previously keratinocyte cultures.

## Author's contribution

FMB performed experiments with NIH3T3, keratinocytes and smooth muscle cells, and CBS with HCT-8, B16F10 and BALB/MK cells. DT and AKC performed purification and characterization of DOKEB. TRM tested analogs of BS in BALB/MK and BALB/MK/LBmSN cells. MBM participated in the preparation of the human primary keratinocytes. SWH drafted the manuscript and conducted all experiments.

## Figures and Tables

**Figure 5:**
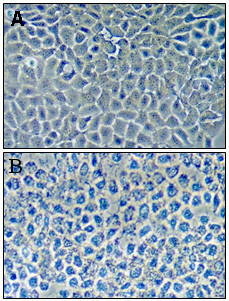
**The lethal effect of the supernatant from the BALB/MK cells transduced with LBmSN **On day 1, BALB/MK cells were seeded on a 6 well plate at 4 × 10^4 ^cells/well, and 1 × 10^6 ^cells of the virus producing cell lines PA317/LBmSN and PA317/LXSN were seeded in 25 cm^2 ^flasks. On day 2, the media of the PA317 cell clones were replaced with the EMEM supplemented with 10 % FBS and without EGF. On day 3, the media of PA317 cells containing virus were harvested, centrifuged at 10,000 rpm per 1 min in Eppendorf centrifuge. The media of BALB/MK cells were replaced by 2 ml of virus solution and complemented with 5 ng/ml of EGF and 8 μg/ml of Polybrene. In parallel, 1 × 10^4 ^BALB/MK cells were seeded on a 24 well plate. On day 4, the media were replaced by fresh ones. On day 5, the medium of BALB/MK cells was replaced by 0.5 ml of the medium of the LBmSN transduced BALB/MK cells, which was previously centrifuged at 10,000 rpm/ 1 min. After 7 days, the cells were photographed (X 200). The results are representative of at least three experiments, which gave essentially the same results. (A) The BALB/MK cells incubated with supernatant of BALB/MK cells transduced with LXSN; (B) The BALB/MK cells incubated with supernatant of BALB/MK cells transduced with LBmSN.

**Table 1:** The effect of cell density on inducing death

Cell concentration	Time till cell death^1 ^(days)
1.0 × 10^3^	8 (0)
5.0 × 10^3^	7 (1)
1.0 × 10^4^	7 (0)
1.5 × 10^4^	6 (1)
2.0 × 10^4^	5 (1)
4.0 × 10^4^	5 (1)

On day 1, 1 × 10^5 ^BALB/MK cells and 1 × 10^6 ^PA317/LBmSN cells were seeded in 25 cm^2 ^flasks. On day 2, the medium from the PA317/LBmSN cells was replaced with EMEM supplemented with 10 % FBS and without EGF. On day 3, the medium of the PA317/LBmSN cells containing virus was harvested, centrifuged at 10,000 rpm per 1 min in an Eppendorf centrifuge. The medium of the BALB/MK cells was replaced with 4 ml of the virus solution and complemented with EGF and Polybrene at a final concentration of 5 ng/ml and 8 μg/ml respectively. On day 4, the cells were trypsinized and seeded in the 24 well plate at the indicated number of cells containing 8 μg/ml BS.

^1 ^Results are the means ± S.D. of three experiments.

**Table 2:** Effect of the BALB/MK cells expressing *bsrm *upon BALB/MK cells

BALB/MK	BALB/MK/LBmSN	BS^1^	EFFECT
+	+	-	D
+	+	+	L
-	+	-	D
-	+	+	L
+	-	-	L

Both cell lines, BALB/MK and BALB/MK/LBmSN, were seeded in a 24 well plate at 1 × 10^4 ^of each one/well or 2 × 10^4 ^cells of one cell line/well. Two days later, the media were replaced by fresh ones, and after 6 days the cells were stained with Coomassie Blue for microscopic analysis. The results are representative of at least two experiments, which gave essentially the same results.

^1 ^[BS] = 8 μg/ml

D = dead

L = alive

+ = presence

- = absence

**Table 3:** Comparison of the vectors LBmSN and LBSN upon BALB/MK and NIH3T3 cells

	BALB/MK		NIH3T3	
	BS^1^	No antibiotic	BS	No antibiotic

LBmSN	L	D	L	L
LBSN	L	L	L	L
No vector	D	L	D	L

The experiment was carried out as described in the legend of the Figure 1. After 8 days of the transduction the cells were stained with Coomassie Blue for microscopic analysis. The results are representative of three independent experiments, which gave essentially the same results.

^1 ^[BS] = In the media of the cells transduced with LBmSN and LBSN were added 8 μg/ml and 2 μg/ml of BS respectively, because the cells transduced with LBSN do not support concentrations of BS higher than 2 μg/ml [8].

D = dead

L = alive
